# Goat pasteurellosis: serological analysis of circulating *Pasteurella* serotypes in Tanqua Aberegelle and Kola Tembien Districts, Northern Ethiopia

**DOI:** 10.1186/s13104-018-3606-0

**Published:** 2018-07-17

**Authors:** Guash Abay Assefa, Mulalem Zenebe Kelkay

**Affiliations:** 1 Abergelle Agricultural Research Center, P.O. Box 44, Abi Adi, Ethiopia; 2 Shire-Maytsebri Agricultural Research Center, P.O. Box 81, Shire, Ethiopia

**Keywords:** Goat, Kola Tembien, Pasteurella, Serotyping, Tanqua Abergelle

## Abstract

**Objectives:**

A cross-sectional study was employed with the aim to explore the serological status of goats; we evaluated the presence of serum antibodies of the circulating serotypes of the genus *Pasteurella*. A total of 124 serum samples were collected from randomly selected goats and subsequently serotyped using indirect haemagglutination test.

**Results:**

In the current study, the overall prevalence of pasteurellosis in goats was 31.4%. Additionally, a total of eight serotypes of *Pasteurella* were serotyped. It is evident that 25% out of 124 sampled animals were found infected by four or more circulating serotypes and 6.4% animals were also found positive for all serotypes. Accordingly, the prevalence of *Pasteurella multocida* serotype A were 16.9%, *Mannheimia haemolytica* serotype A1 26.6%, *M. haemolytica* serotype A2 18.5%, *M. haemolytica* serotype A7 16.1%, *Bibersteinia trehalosi* serotype T3 20.9%, *B. trehalosi* serotype T4 21.7%, *B. trehalosi* serotype T10 27.4%, and *B. trehalosi* serotype T15 was 25.8%. Therefore, although there has been vaccination campaign with monovalent vaccine *P. multocida* type A, the diseases still exerts negative impacts through death of goats to smallholder farmers. Therefore, to control the disease the government should provide multivalent vaccine of the above serotypes.

**Electronic supplementary material:**

The online version of this article (10.1186/s13104-018-3606-0) contains supplementary material, which is available to authorized users.

## Introduction

In Ethiopia, goat pasteurellosis caused by *Pasteurella* is more prevalent and where outbreak occur lead to high mortality. *Pasteurella multocida* or *Mannheimia haemolytica* are the main causes for respiratory pasteurellosis in sheep and goats of all age groups [1]. It can be particularly devastating in young animals. It is a common cause of high morbidity and mortality in kids, especially in those that have not received adequate colostrum. The disease appears to occur most often in animals that have undergone recent stress such as transportation, weaning, or commingling with animals from unrelated farms [2]. Development of carrier status or latent infections plays a critical role in the epidemiology of the diseases [3].

Even though there had been research works done on animal health in Tigray region, there is no evidence that shows the clear picture of the serotypes causing pasteurellosis in the study districts. Based on the information obtained from the districts veterinary services large and small ruminant pasteurellosis is one of the major health problems and the vaccination is done by purchasing the recommended vaccine from the National Veterinary Institute (NVI) which is developed from only *P. multocida* type A, resulting poor vaccine efficacy. Although there is a vaccination campaign once each year, vaccination is irregular because of inadequate vaccine supply and lack of farmer awareness. Hence, this research study was conducted to know the existing serotypes from randomly selected goats.

## Main text

### Materials and methods

#### Study area

**Fig. 1:**
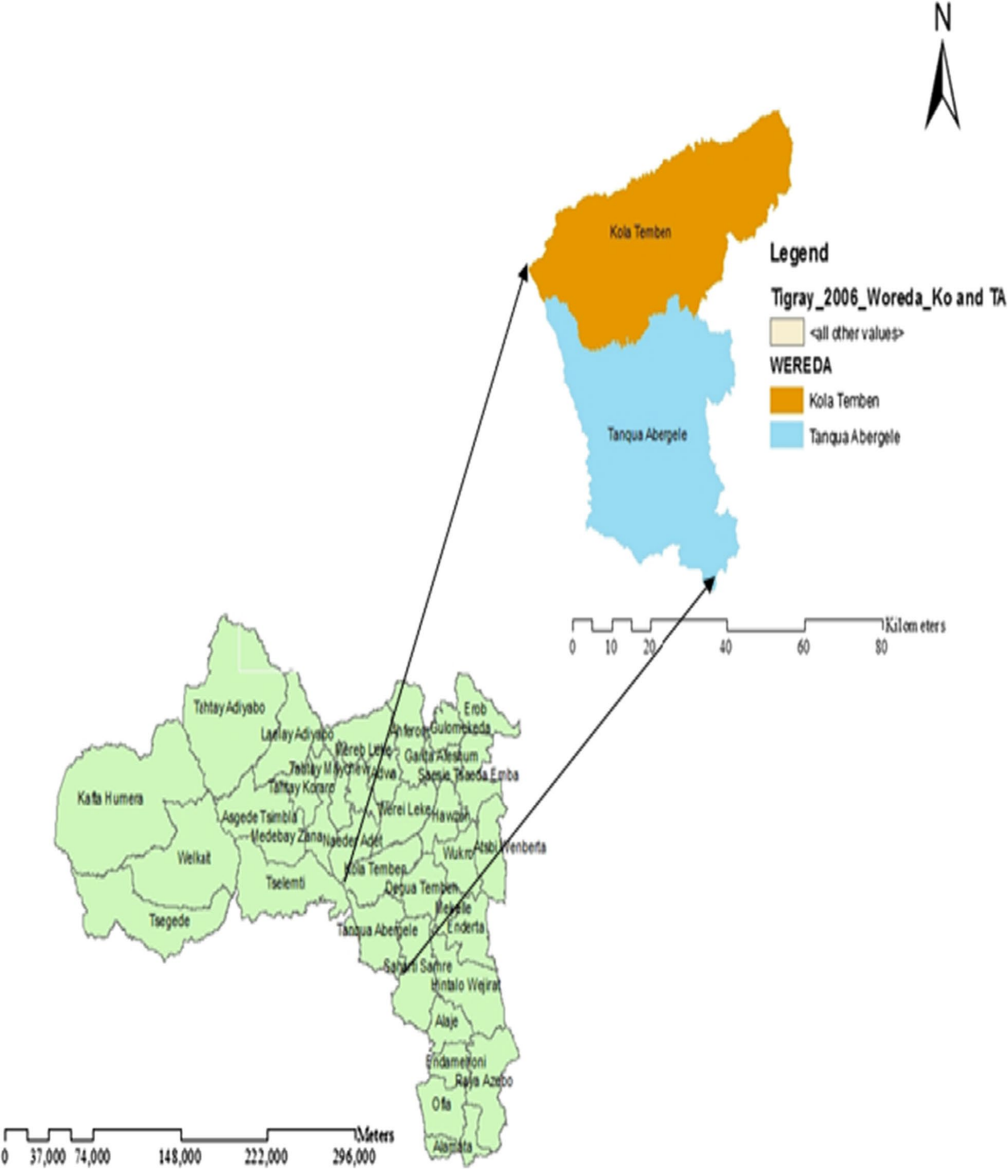
Topographic map of Tanqua Abergele and Kola Tembien

As shown in Fig. 1, the study was conducted in Kola Tembien, and Tanqua Abergelle districts for disease investigation and sample collection. The study districts are categorized as hot to warm sub-moist low lands sub-agro ecological zone of the region with an altitude of 1300–1500 m above sea level and the mean annual rainfall ranging from 400 to 600 mm, which is characterized by low, erratic and variable rainfall. The annual temperature ranges from 28 to 42 °C [4].

#### Study methodology

In the current study, a cross sectional study design was used to identify the circulating serotypes of *Pasteurella.* Individual animals were selected randomly with no prior vaccination against the disease in both study districts regardless of their age and sex. Blood samples (5 ml) were withdrawn aseptically from the external jugular vein using plain vacutainer tubes. For this purpose, a total of 124 blood samples were collected and subsequently allowed to clot, and then serum was separated immediately by centrifugation at 1500*g* for 10 min. Serum samples were stored in refrigerator at a temperature of − 20 °C and were taken to NVI for serological identification using IHAT. Each serum samples were serotyped using the reference serotypes available in NVI using IHAT. The objective of indirect haemagglutination test is to detect the presence of antibody of *Pasteurella* and *Manhaemia* in the serum sample. The IHAT antibody titres of all the samples were recorded in comparison with the positive and negative controls. Positive results were taken if the level of antibody titer was greater than 1:10 (Additional file 1).

#### Sample size determination

The sample size for the study was determined according to the formula given by [5] for the purpose of random sampling method. A 5% absolute precision and 95% confidence interval was used to determine the sample size. An expected prevalence of 8.46% was taken to determine the maximum sample size [6]. Accordingly, 119 animals were used for the present study.

$$N = \frac{{1.96^{2} \;P_{\exp } (1 - P_{\exp } )}}{{d^{2} }}$$

where n is total sample size; d is absolute precision; and P_exp_ is expected prevalence.

#### Data analysis

Data were entered into Microsoft excel and analyzed using descriptive statistics so as to compare the circulating serotypes in terms of frequency.

### Result

#### Prevalence of *Pasteurella* and *Manhaemia* serotypes

Infection in goat due to *Pasteurella* species is the main contributor for economical loss in the study districts. The prevalence of pasteurellosis serotypes in the study districts is given in Table 1.

**Table 1:** Prevalence of *Pasteurella* serotypes in the study districts

Serotype name	Positive cases	Percent
*Pasteurella multocida* serotype A	21	16.9
*Mannheimia haemolytica* serotype A1	33	26.6
*Mannheimia haemolytica* serotype A2	23	18.5
*Mannheimia haemolytica* serotype A7	20	16.1
*Bibersteinia trehalosi* serotype T3	26	20.9
*Bibersteinia trehalosi* serotype T4	27	21.7
*Bibersteinia trehalosi serotype T10*	34	27.4
*Bibersteinia trehalosi* serotype T15	32	25.8

Out of the 124 goats sampled, the prevalence of seropositive goats was 16.9% (21/124) for *P. multocida*, 28.2% (35/124) for *M. haemolytica*, and 31.4% (39/124) for *Bibersteinia trehalosi*. The overall prevalence of pasteurellosis in goat in the study districts were 31.4% (39/124). The study shown that a higher prevalence of *M. haemolytica* (28.2%) and *B. trehalosi* (31.4%).

Of the 124 tested animals 31 (25%) animals were infected by four or more serotypes. Additionally, 8 (6.45%) animals, 7 (5.6%) animals, 8 (6.45%) animals, 4 (3.2%) animals, 5 (4.0%) animals were infected by eight, seven, six, five, and four serotypes respectively.

Overall, the most prevalent serotypes identified were *Pasteurella trehalosi serotype T10*, *M. haemolytica* serotype A1, *B. trehalosi* serotype T15, *B. trehalosi* serotype T4, *B. trehalosi* serotype T3, and *M. haemolytica* serotype A2.

### Discussion

Pasteurellosis is among the most important infectious diseases of goats in the study districts that causes huge economical loss through death. In these study districts, the disease is mostly associated to stress conditions such as drought, heat, and usually commence during the beginning of rain fall.

The species are commensally resident in the upper respiratory tract of healthy ruminants and are capable of causing infection when animals immunity become compromised [7].

In the present study, the dominant pasteurella species encountered were *B. trehalosi* (31.4%), and *M. haemolytica* (28.2%). Therefore, these pasteurella species are the dominant causative agents for pasteurellosis infection in the study districts. The results published here are in agreement with those of other authors [8–10]. In contrast to our study *M. haemolytica* in goat was not dominant in different studies [11]. Our finding shows higher prevalence (31.4%) in pasteurellosis infection. [10] Have found a prevalence of 21.9% which is below to our study. In the current study the prevalence is higher as compared to previous report (8.46%) by [6] in the same study districts. This high prevalence in our study may be due to high environmental stress factors (heat, drought, feed and water shortages) in the study districts since 2015. Transportation, viral infections, drought, overcrowding, housing of neonates and weaned animals together and other stressful conditions predispose animals to *M. haemolytica* and *P. multocida* infection [12, 13]. *M. haemolytica* serotype A1 in the study district were isolated with the prevalence of 26.6% which is higher than the findings of [14] which they found with the prevalence of 19% in Bishoftu district.

In our findings, a combination of more than two sero-types infection on a single animal has been observed. Similar to our findings, [15] has been showed mixed infection of serotypes on a single animal in Tigray region districts.

### Conclusions

In conclusion, higher prevalence of pasteurellosis infection was found in the study districts. About eight serotypes of *Pasteurella* species were serotyped. Therefore, multivalent *Pasteurella* vaccine or a single vaccine of the identified serotypes should be developed and regular vaccination with full area coverage should be practiced.

## Limitations

This research was only conducted to know the serotypes of *Pasteurella* by using IHAT. Further isolation and characterization of the serotypes has not been conducted.

## Additional file

**Additional file 1.** The data collected (serum) for this study.

## Abbreviations

IHAT: indirect haemagglutination test
NVI: National Veterinary Institute
ml: milliliter
°C: degree centigrade
